# High rates of de novo 15q11q13 inversions in human spermatozoa

**DOI:** 10.1186/1755-8166-5-11

**Published:** 2012-02-06

**Authors:** Òscar Molina, Ester Anton, Francesca Vidal, Joan Blanco

**Affiliations:** 1 Unitat de Biologia Cel·lular (Facultat de Biociències). Universitat Autònoma de Barcelona. 08193-Bellaterra (Cerdanyola del Vallès), SPAIN

**Keywords:** Low Copy Repeats, Non-allelic Homologous Recombination, 15q11q13 Inversions, Spermatozoa, Fluorescence *in situ *Hybridization

## Abstract

Low-Copy Repeats predispose the 15q11-q13 region to non-allelic homologous recombination. We have already demonstrated that a significant percentage of Prader-Willi syndrome (PWS) fathers have an increased susceptibility to generate 15q11q13 deletions in spermatozoa, suggesting the participation of intrachromatid exchanges. This work has been focused on assessing the incidence of *de novo *15q11q13 inversions in spermatozoa of control donors and PWS fathers in order to determine the basal rates of inversions and to confirm the intrachromatid mechanism as the main cause of 15q11q13 anomalies.

Semen samples from 10 control donors and 16 PWS fathers were processed and analyzed by triple-color FISH. Three differentially labeled BAC-clones were used: one proximal and two distal of the 15q11-q13 region. Signal associations allowed the discrimination between normal and inverted haplotypes, which were confirmed by laser-scanning confocal microscopy.

Two types of inversions were detected which correspond to the segments involved in Class I and II PWS deletions. No significant differences were observed in the mean frequencies of inversions between controls and PWS fathers (3.59% ± 0.46 and 9.51% ± 0.87 vs 3.06% ± 0.33 and 10.07% ± 0.74). Individual comparisons showed significant increases of inversions in four PWS fathers (*P *< 0.05) previously reported as patients with increases of 15q11q13 deletions.

Results suggest that the incidence of heterozygous inversion carriers in the general population could reach significant values. This situation could have important implications, as they have been described as predisposing haplotypes for genomic disorders. As a whole, results confirm the high instability of the 15q11-q13 region, which is prone to different types of *de novo *reorganizations by intrachromatid NAHR.

## Background

The human genome has been proven to be a highly dynamic structure, showing a great number of structural and copy-number variations [1]. Four major mechanisms contribute to the genesis of variations: non-allelic homologous recombination (NAHR), non-homologous end joining (NHEJ), fork stalling and template switching (FoSTeS) and retrotransposition [1]. The presence of segmental duplications or low-copy repeats (LCR) throughout the human genome plays a significant role in the formation of variation through NAHR [2,3]. LCRs are DNA fragments longer than 1 Kb in size which share more than 90% of sequence identity between paralogous copies [4]. They represent 5% of the human genome, and their interspersed nature and sequence identity provide a substrate for NAHR [5].

**Figure 1:**
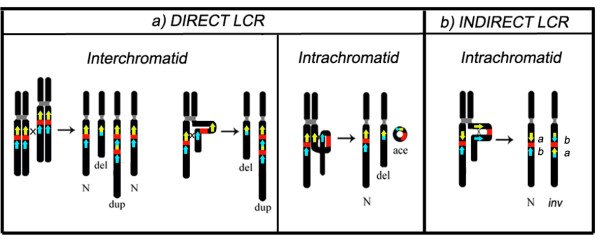
**Products generated by NAHR according to the LCR orientation and the chromatids involved**. (N = normal; del = deletion; dup = duplication; ace = acentric; inv = inversion).

Different stable products can be produced by NAHR according to the orientation of the LCR and the number of chromatids involved in the event. Complementary deletions and duplications can be generated by interchromatid NAHR involving direct LCRs, deletions will be the only resulting product of intrachromatid NAHR also involving direct LCRs. Inversions will be generated via intrachromatid NAHR if LCRs are arranged in an indirect orientation (Figure 1). While deletions and duplications are usually related to altered phenotypes, most inversions are considered as being polymorphic variants with no apparent phenotypic effects for the carriers. This fact, together with the lack of high-throughput methods currently available for the detection of submicroscopic inversions, leads to an underestimation of the true amount of their real occurrence in the human genome [6]. Nevertheless, it has been postulated that they can increase the likelihood of secondary rearrangements leading to recurrent genomic disorders in the offspring [7] (Table 1).

**Table 1:** Examples of polymorphic inversions and the related genomic disorders reported to be caused as secondary rearrangements.

INVERSION	SIZE (Mb)	GENOMIC DISORDER/REARRANGEMENT	REFERENCE
inv(3)(q29)	1.9	3q29 microdeletion syndrome	[29]
inv(5)(q35)	1.3	Sotos syndrome	[30]
inv(7)(q11.23)	1.8	Williams-Beuren syndrome	[31]
inv(8)(p23)	4.7	8p23 microdeletion syndrome	[32]
inv(15)(q11q13)	4	Angelman syndrome	[12]
inv(15)(q13.3)	2	15q13.3 deletion syndrome	[33]
inv(17)(q12)	1.5	RCAD syndrome	[29]
inv(17)(q21.31)	0.9	17q21.21 deletion syndrome	[34]

The human 15q11-q13 region is a highly dynamic segment involved in different rearrangements by NAHR. This region is flanked by five complex LCRs (LCR15-1, LCR15-2, LCR15-3, LCR15-4 and LCR15-5), clustering most of the breakpoints involved in chromosome reorganizations [8]. LCRs of the 15q11-q13 region are built by duplications of the *HERC2 *gene/pseudogene which form blocks called END-repeats that are oriented both in direct or indirect orientations [9]. Two major types of deletions have been reported: Class-I deletions involve a 6.6-Mb region delimitated by two breakpoints localized within the LCR15-1 and LCR15-3, and Class-II deletions, which have the proximal breakpoint within the LCR15-2 and the distal one in the LCR15-3, leading to a loss of 5.3 Mb of genetic material [10]. It is well known that paternal 15q11q13 deletions are the major cause of Prader-Willi syndrome (PWS; OMIM 176270) [11], while maternal deletions cause Angelman syndrome (AS; OMIM 105830) [11].

Inversions of the 15q11-q13 region have been indirectly related to the occurrence of genomic disorders. In particular, Gimelli et al. (2003) [12] reported a 5.3-Mb heterozygote inv(15)(q11q13) (corresponding to the Class-II deletion segment) in a significant proportion of mothers with Angelman syndrome-affected children. Based on these findings, they suggested the existence of haplotypes at risk for the generation of secondary rearrangements (deletions and/or duplications) as was previously described in the 7q11.23 region [13]. Strikingly, this inversion was also observed in heterozygosis in 9% of the control population. Other NAHR-based chromosomal rearrangements have been reported in this region that emphasize its instability: duplications [14], supernumerary marker chromosomes [15], triplications [16] or partial uniparental disomies [17].

Our group has recently reported that some PWS fathers produce significantly increased frequencies of spermatozoa carrying *de novo *15q11q13 deletions, suggesting the presence of predisposing haplotypes for intra-chromatid NAHR events [18]. As intra-chromatid NAHR events could also generate inversions, in the present work we have analyzed the frequency of *de novo *15q11q13 inversions in spermatozoa from control donors and PWS fathers. The aim of this work is: 1) to determine the basal rate of *de novo *15q11q13 inversions, and 2) to investigate whether the PWS fathers with an increase of deletions also show increases of inversions.

## Results

### Clone selection and positioning

**Figure 2:**
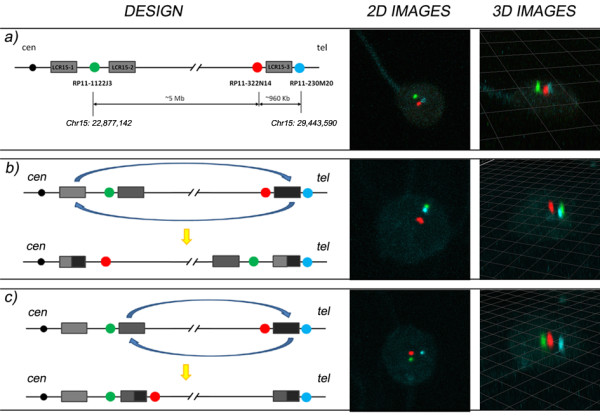
**Spermatozoa classification regarding signal distribution**. Examples of two- and three-dimensional images of the same sperm nucleus are showed. a) Normal haplotype, b) Type-1 inversion haplotype and c) Type-2 inversion haplotype.

Three BAC clones were selected using the resources of the Genome Browser database (UCSC Assembly; February 2009) [19] for triple-color FISH experiments on decondensed sperm-nuclei: one proximal BAC clone (RP11-1122J3) mapping between the LCR15-1 and LCR15-2, and two distal clones (RP11-322N14 and RP11- 230M20) on either side of the LCR15-3 (Figure 2a).

The positions of the selected probes were verified on metaphase spreads. Every single probe showed specific signals on the pericentromeric area of chromosome 15 (identified by DAPI banding), and no cross-hybridization was observed.

### Experimental design

A minimum of 1,000 informative sperm nuclei were analyzed per sample (controls and PWS fathers). The following criteria of analysis were strictly used:

1. Only spermatozoa with a well-defined boundary were evaluated. Overlapping spermatozoa were discarded from the count.

2. Only spermatozoa with a clear distribution of two associated signals (in close proximity or overlapped) and one separated signal were considered informative. The separated signal must be separated from the others by at least a two-fold longer distance compared with the separation of the associated signals.

Our design implies that two probes would potentially change their positions (RP11-1122J3 and RP11-322N14) while the third probe remains in place (RP11-230M20). This allowed us to unequivocally discriminate between normal and inverted haplotypes. According to the distribution of signals in the informative sperm nuclei, the following genotypes were assigned:

• Normal (Figure 2a): spermatozoa displaying an association of the two distal signals (orange and blue) and the proximal signal (green) located apart from the others.

• Type-1 inversion (Figure 2b): spermatozoa displaying the proximal signal (green) associated with the most distal signal (blue), and the orange signal being located apart.

• Type-2 inversion (Figure 2c): spermatozoa displaying the proximal signal (green) associated with the orange signal, and the blue signal being located apart.

The presence of normal and inverted haplotypes was confirmed by confocal laser- scanning microscopy (Figure 2).

### Sperm-FISH results

The hybridization efficiency of every single probe in sperm nuclei was higher than 98%.

The mean frequencies of informative nuclei in both controls and PWS populations were higher than 70% (71.45% and 73.78%, respectively) (Tables 2 and 3).

**Table 2:** Sperm-FISH results in control donors

CASES	Age	Total	Informative (%)	Haplotypes^a^		
				
				Normal	Type-1 inv	Type-2 inv
**C-1**	26	1416	1015 (71.68)	873 (86.01)	54 (5.32)	88 (8.67)
**C-2**	24	1479	1061 (71.74)	932 (87.84)	41 (3.86)	88 (8.29)
**C-3**	25	1494	1018 (68.13)	842 (82.71)	45 (4.42)	131 (12.87)
**C-4**	23	1435	1063 (74.08)	937 (88.15)	56 (5.27)	70 (6.59)
**C-5**	36	1343	1012 (75.35)	862 (85.18)	45 (4.45)	105 (10.38)
**C-6**	28	1488	1024 (68.82)	834 (81.44)	46 (4.49)	144 (14.06)
**C-7**	50	1663	1051 (63.20)	960 (91.34)	23 (2.19)	68 (6.47)
**C-8**	50	1461	1005 (68.79)	852 (84.78)	28 (2.79)	125 (12.44)
**C-9**	42	1210	1003 (82.89)	912 (90.93)	13 (1.29)	78 (7.78)
**C-10**	26	1490	1040 (69.80)	943 (90.67)	19 (1.83)	78 (7.50)

**% ± SEM**			71.45% ± 1.66	86.91% ± 1.10	3.59% ± 0.46	9.51% ± 0.87

^a ^Number and percentage of informative nuclei

**Table 3:** Sperm-FISH results in PWS fathers

CASES	Age	Total	Informative (%)	Haplotypes^a^		
				
				Normal	Type-1 inv	Type-2 inv
**PW-1**	41	1309	1012 (77.31)	802 (79.35)	58 (5.73)*	152 (15.02)*
**PW-2**	35	1278	1313 (79.26)	901 (88.94)	21 (2.07)	91 (8.98)
**PW-3**	44	1274	1017 (79.83)	929 (91.35)	21 (2.06)	67 (6.59)
**PW-4**	35	1373	1065 (77.57)	919 (86.29)	35 (3.29)	111 (10.42)
**PW-5**	30	1604	1045 (65.15)	874 (83.64)	38 (3.63)	133 (12.73)*
**PW-6**	33	1375	1008 (73.31)	903 (89.58)	26 (2.58)	79 (7.84)
**PW-7**	47	1465	1060 (72.35)	852 (89.81)	22 (2.08)	86 (8.11)
**PW-8**	50	1406	1048 (74.54)	907 (86.54)	24 (2.29)	117 (11.16)
**PW-9**	60	1462	1095 (74.90)	1021 (93.24)	15 (1.37)	59 (5.39)
**PW-10**	60	1321	1014 (76.76)	831 (91.95)	42 (4.14)	141 (13.90)*
**PW-11**	42	1313	1010 (76.92)	847 (83.86)	43 (4.26)	120 (11.88)
**PW-13**	53	1509	1063 (70.44)	856 (80.53)	54 (5.08)	153 (14.39)*
**PW-14**	55	1500	1015 (67.67)	929 (91.53)	22 (2.17)	64 (6.31)
**PW-15**	47	1357	1007 (74.21)	881 (87.49)	44 (4.37)	82 (8.14)
**PW-16**	44	1462	1014 (69.36)	886 (87.38)	17 (1.68)	111 (10.95)
**PW-17**	-	1494	1059 (70.88)	937 (88.48)	23 (2.17)	99 (9.35)

**% ± SEM**			73.78% ± 1.05	86.87% ± 1.02	3.06% ± 0.33	10.07% ± 0.74

^a ^Number and percentage of informative sperm nuclei

* Statistically significant increases (*P *< 0.05).

A total of 10,292 informative sperm nuclei from the control population were analyzed (Table 2). The mean frequency of Type-1 inversions was 3.59%, ranging from 1.29% to 5.32%, with a standard error of the mean (S.E.M) of 0.46%. The mean frequency of Type-2 inversions was 9.51% (± 0.87% SEM), ranging from 6.47% to 14.06%.

In fathers of PWS individuals, a total of 16,545 informative sperm nuclei were analyzed (Table 3). The mean frequency of Type-1 inversions was 3.06% (± 0.33% SEM), ranging from 1.37% to 5.73%. The mean frequency of Type-2 inversions was 10.07% (± 0.73% SEM), ranging from 5.39% to 15.02%.

**Figure 3:**
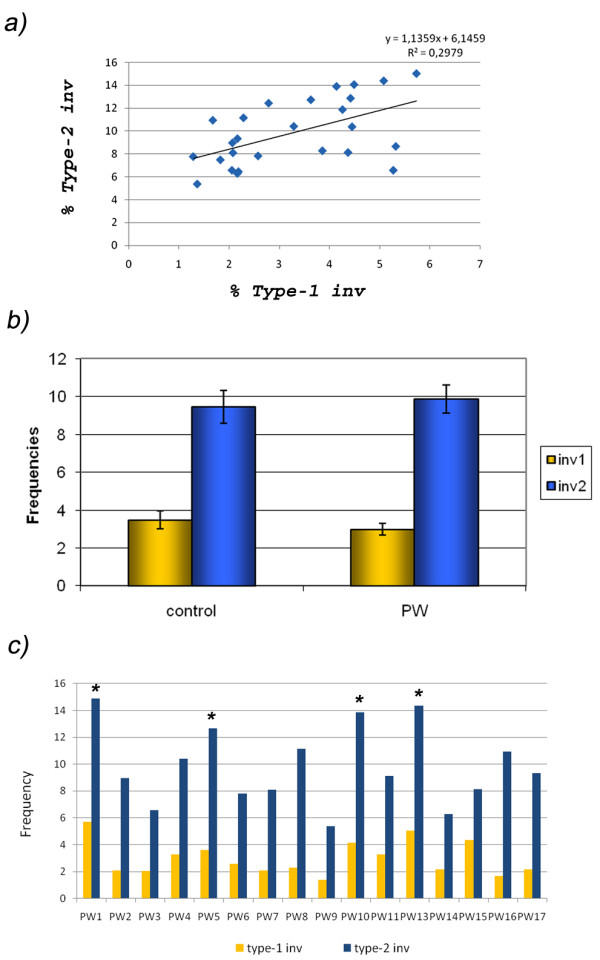
**a) Correlation between the frequencies of Type-1 and Type-2 inversions, b) Mean frequencies of 15q11q13 inversions in control donors and PWS fathers**. Bars represent the standard error of the mean (SEM), c) Frequencies of Type-1 and Type-2 inversions observed in every single PWS father. Asterisks show cases with significant higher incidences of inversions.

Frequencies of Type-2 inversions were significantly higher than the frequencies of Type-1 inversions in both the control population (*P *= 0.005) and in the PWS fathers (*P *= 0.0001). Moreover, a significant correlation was observed between the frequencies of Type-1 and Type-2 inversions (r = 0.55; *P *= 0.004) (Figure 3a).

No significant differences were observed for either the frequencies of Type-1 or Type-2 inversions between the PWS fathers and the controls (*P *> 0.05) (Figure 3b). Nevertheless, individual comparisons showed higher incidences of inversions in four PWS fathers due to increases of Type-2 inversions: PW-5, PW-10 and PW-13 (*P *< 0.05), or increases in both types of inversions: PW-1 (P < 0.05) (Table 3) (Figure 3c). It deserves to be mentioned that significant increases of 15q11q13 deletions were previously reported in all these four individuals [18]. No significant correlation was observed between the frequencies of 15q11q13 inversions and age (*P *> 0.05).

When considering the frequency of the different stable products resulting from intrachromatid NAHR events, that is, inversions and deletions (data previously reported [18]), no correlation was observed in either the control population (*P *= 0.121) or in the PWS fathers. However, a tendency to correlate (r = 0.69; *P *= 0.062) was observed in the PWS fathers.

## Discussion

### Experimental design

Sperm-FISH analyses have been widely used for cytogenetic studies in spermatozoa [20] and have been mainly focused on evaluating the genetic reproductive risk of carriers of abnormal karyotypes [21] and infertile patients [22]. Furthermore, and more recently, we have demonstrated the reliability of this methodology to assess the rates of deletions and duplications in spermatozoa [18]. In all of these approaches, the chromosomal constitution of the sperm nuclei is inferred by the evaluation of the presence or absence of a given FISH signal.

In the present work, the potential of sperm-FISH to detect the inversions has been evaluated. Our results confirm that this approach is feasible and realistic if an accurate experimental design is used, taking into consideration the relation between the genetic distances and the physical distances of FISH signals in the interphase nucleus.

Our experimental design has led to the observation of two types of signal distributions: two plus one (2+1) and associations of three. As the images of the three signals in association could be the consequence of chromatin loops resulting from high-order packaging of interphase DNA, all of the nuclei showing this distribution were classified as uninformative and were discarded for haplotype evaluation. The 2+1 distributions were assigned to three different haplotypes: normal, Type-1 and Type-2 inversions. In any case, the two associated probes were always located closer than 1 Mb and the probe apart was separated by a distance longer than 5 Mb. It is well-known that the separation of interphase FISH signals increases as the genomic distance between the probes becomes larger, and this correlation is consistent for genomic separations up to 2 Mb [23]. As a consequence, the three signals in our experimental design will appear as specific distributions of 2 associated dots plus 1 separated dot.

### Types of 15q11q13 inversions

The results obtained point to the existence of two types of inversions that involve the segments corresponding to the reported Class-I and Class-II 15q11q13 deletions. Our data is in agreement with the existence of the 15q11q13 inversions between the LCR15-2 and 3 (Type-2) previously described by other authors [12]. Furthermore, our experimental approach allowed us to describe a new inversion involving the segment between LCR15-1 and 3 (Type-1 inversion). As in the case of deletions, the most frequent inversion was the Type-2 inversion. Altogether, these data suggest that the LCR15-2 could harbor longer stretches of homology than LCR15-1 with the LCR15-3, making the first more susceptible to NAHR events as was previously inferred by Makoff and Flomen [9]

### Susceptibility to generate 15q11q13 inversions

Unexpectedly high rates of 15q11q13 inversions were found in spermatozoa from the two populations studied. Two pieces of evidence indicate that these inversions are generated *de novo*: 1) Since the frequency of inversions was not close to 50% in any case, the possibility of having any constitutional heterozygote inversion carrier can be ruled out. 2) Another possibility is the presence of a mosaicism for the inversion restricted to the germ line. Two pieces of data make such a possibility unlikely. First, inversions are considered to be a predisposing haplotype that increases the risk of secondary rearrangements such as deletions and duplications. While the incidence of inversion was consistently high in all control and PWS fathers, most of them did not show increases of deletions. Second, if mosaicism is present in testicular tissue, we would expect that the degree would be different among individuals generating different percentages of sperm inversions. Instead, as mentioned above, the percentage of sperm inversions was consistent (Tables 2 and 3).

The high rate of 15q11q13 inversions in spermatozoa suggests that a certain percentage of heterozygous carriers are present among the general population. Taking into account the frequency of sperm inversions observed in the present study (Type-1 and Type-2 inversions: 13.1%) and assuming that the frequency of these inversions would be the same in oocytes, we can infer that the frequency of heterozygous carriers in the general population would be 22.6% (2 × 13/100 × 87/100). These results are in agreement with the 9% of inv(15)(q11q13) heterozygous carriers (4 out of 44 individuals) found in the control population assessed by Gimelli et al.(2003) [12]. This situation has already been described in population studies analyzing other regions with similar features such as 7q11.23 and 8p23.1, where inversions were observed in 5.8% and 79%, respectively [24,25]. In this sense, it seems likely that the frequency of inversions in regions with a genomic architecture characterized as being flanked by LCR could be as high as the one described in the 15q11-q13 region, and this would strengthen the hypothesis that the frequencies of inversions are underestimated within the great deal of structural variants of the human genome [6].

This situation might have important implications at a population level. As was previously suggested, the presence of an inverted chromosome in heterozygosity originates an unpaired region at pachytene making the region prone to misalignment and NAHR. Thus, the risk for a secondary rearrangement affecting the offspring could be increased [6,12,13]. Consequently, heterozygote carriers of any of these types of inversions could have an increased risk of transmission of 15q11q13 deletions in descendants.

### Intrachromatid NAHR is the major NAHR mechanism

Results obtained in the present work showed higher frequencies of 15q11q13 inversions than the frequencies of deletions and duplications previously reported in controls and PWS fathers [18], thus indicating that intrachromatid NAHR is the most frequent mechanism. Some PWS fathers showed significantly increased rates of inversions, as compared with the control population (Table 3). Strikingly, all of them were previously reported to have significant increases of 15q11q13 deletions [18]. These data point out the important participation of intrachromatid NAHR in the generation of 15q11-q13 anomalies. As a whole, our results suggest that individuals at risk (those showing significant increases of 15q11q13 anomalies in spermatozoa) have a disrupted intrachromatid NAHR mechanism, as only the products derived from this mechanism are significantly increased.

Since no constitutional heterozygous carriers of inversions were detected in any of the subjects analyzed, and different outcomes were found to be increased in spermatozoa of some PWS fathers (15q11q13 deletions or both 15q11q13 deletions and inversions), differences in the genomic architecture of the LCRs flanking this region might exist. Some authors have reported structural variation in the LCRs flanking some regions involved in genomic disorders [26,27]. They suggested that some specific haplotypes within these LCRs could predispose those regions to misalignment between paralogous copies and thus predispose the region to NAHR, increasing the risk of transmission of genomic disorders. Our results suggest that, in fact, differences in the genomic architecture of the LCR15s predispose these individuals to intrachromatid NAHR, thus producing different rearrangements. Some individuals would show haplotypes with longer stretches of direct homology between paralogous LCRs, thus increasing the rates of deletions in spermatozoa, whereas other individuals might have haplotypes with higher degrees of direct and inverted homologies with their own paralogous copies, thus increasing both the rates of *de novo *deletions and inversions in spermatozoa. In this sense, the availability of methodologies that enable studying the genomic architecture of specific LCRs would allow for identifying possible predisposing haplotypes that would explain these differences and that would potentially increase the risk of transmission of 15q11q13 anomalies.

## Conclusions

The high incidence of de novo 15q11q13 inversions in spermatozoa indicates that the incidence of heterozygous inversion carriers in the general population could reach significant values. As a whole, results confirm the high instability of the 15q11-q13 region, which is prone to different types of de novo reorganizations, mainly generated by intrachromatid NAHR events.

## Methods

### Biological samples

Semen samples from 10 control donors aged between 24 and 50, and 16 PWS fathers from 32 to 60 years of age were obtained. Donors were volunteers recruited from the general population. All subjects had normal karyotypes and were normozoospermic. To our knowledge, none of them had been exposed to genotoxic agents, and no history of chemotherapy, radiotherapy or chronic illness was recorded.

All subjects gave their informed consent in writing to participate in the study and the protocol was approved by the Institutional Ethics Committee of the Universitat Autònoma de Barcelona.

### Slide preparation

Samples were processed as described previously by our group [28]. Briefly, the sperm fraction was resuspended in hypotonic solution (0.075 M KCl) for 30 minutes at 37°C and fixed in methanol:acetic acid (3:1). Spermatozoa were spread on a slide and kept at -20°C until processed.

### Probes

Three BAC clones were selected using the resources of the Genome Browser database (UCSC Assembly; February 2009). All clones were obtained from the Children's Hospital Oakland Research Institute, BACPAC resources (Oakland, CA 94609 USA). BAC DNA extraction was performed using the QIAprep Miniprep kit (Qiagen GmbH; Hilden, Germany) following manufacturer's instructions.

BAC clones RP11-1122J3, RP11-322N14 and RP11-230M20 were fluorescently labeled with Spectrum Green-dUTPs (Abbott Molecular; Abbott Park, IL, USA), Spectrum Orange-dUTPs (Abbott Molecular) and DEAC-dUTPs (Perkin Elmer Inc; Boston, USA), respectively, by Nick Translation (Roche; Mannheim, Germany). Probes were mixed with 10 μg of Cot 1 DNA (Invitrogen; Carlsbad, USA), ethanol precipitated and resuspended in hybridization buffer (50% Formamide, 1xSSC and 10% dextran sulphate) (Abbott Molecular).

Probe positions were verified on lymphocyte metaphase chromosomes. Hybridization in spermatozoa was determined by the evaluation of 1,000 sperm nuclei per probe in three different FISH experiments.

### Fluorescence *in situ *Hybridization on sperm (sperm-FISH)

Prior to hybridization, sperm nuclei were decondensed by slide incubation at 37°C in Tris buffer containing 25 mmol/ml dithiothreitol and 1% Triton X-100 for 45 minutes.

A triple-color FISH using the three BAC clones differentially labeled was performed following standard procedures. Briefly, probes were denatured at 80°C for 8 minutes and pre-annealed at 37°C for 15 minutes. Sperm nuclei were denatured at 73°C in 70% formamide in 2xSSC for 5 minutes. Hybridization was carried out by adding 5 μl of the corresponding probe mixture (200 ng of each probe) to the sperm preparation and incubating the slides in a moist chamber at 37°C for 48 hours. Post-hybridization washes were performed in 1xSSC with 0.3% NP-40 at 73°C followed by 2xSSC with 0.1% NP-40 at room temperature, for one minute in each solution. Slides were mounted with antifade solution (Abbott Molecular).

Analyses were performed using an Olympus BX-60 epifluorescence microscope equipped with a triple-band pass filter, and specific filters for Aqua, FITC and Cy3.

A minimum of 1,000 informative sperm nuclei were analyzed per sample.

### Confocal Laser-Scanning Microscopy

To confirm the existence of normal and inverted haplotypes, 80 sperm-nuclei were captured and analyzed using a confocal-laser scanning TCS-SP5 AOBS microscope (Leica Microsystems, Heidelberg Gmbh; Mannheim, Germany).

Spectrum Orange fluorochromes were excited with the 561-nm line of a DPSS laser and observed in the red channel at an emission range of 569-nm to 671-nm. Spectrum Green fluorochromes were excited with the 405-nm line of a diode laser and viewed in the green channel at 502-nm to 551-nm. Finally, DEAC fluorochromes were excited with the 488-nm line of an Argon laser and observed in the blue channel at an emission range of 444-nm to 500-nm. Stacks of 16 to 20 sections every 0.3 μm were acquired.

Image combining and processing were performed with the IMARIS software package version 2.7 (Bitplane AG; Zürich, Switzerland).

### Data Analyses

Data obtained were statistically analyzed using SPSS version 15.0 (SPSS Inc; Chicago, IL, USA) under the advice of the statistical service of the Universitat Autònoma de Barcelona.

To assess the relationship between the two inverted haplotypes, the following analyses were performed:

• The mean frequencies of Type-1 and Type-2 inversions were compared in both populations by a Wilcoxon test.

• A Pearson's correlation test between the frequencies of the two inverted haplotypes was performed.

To assess the susceptibility of the 15q11-q13 region to generate inversions, the following statistical tests were performed:

• Population level: The mean population frequency of 15q11q13 inversions were compared between controls and PWS fathers by means of the Mann-Whitney test.

• Individual level: A Chi-square test comparing the inversion frequencies of every single PWS father with the mean frequency of inversions observed in the control population was performed.

• Age effect: A Pearson's correlation test between the sum of inversions and the age of all subjects was performed.

To assess a possible relationship between the frequency of inversions and the frequency of deletions previously found by our group [18]:

• Spearman's correlations between the frequency of the total inversions (Type-1 + Type-2) and the frequency of deletions were performed for both the control population and the PWS fathers.

Differences and correlations were considered statistically significant when *P *< 0.05.

## Competing interests

The authors declare that they have no competing interests.

## Authors' contributions

OM was responsible for conception and design, acquisition of data, data analysis and interpretation, writing the manuscript and final approval. EA was responsible for revision of data and interpretation and final approval. FV was responsible for conception and design, data analysis and interpretation, writing the manuscript and final approval. JB was responsible for conception and design, data analysis and interpretation, writing the manuscript and final approval.

All authors read and approved the final version of the manuscript.
